# Effect of Heat Treatment on Microstructures and Mechanical Properties of TC4 Alloys Prepared by Selective Laser Melting

**DOI:** 10.3390/ma18174126

**Published:** 2025-09-02

**Authors:** Jian Zhang, Yuhuan Shi, Su Shen, Shengdong Zhang, Honghui Ding, Xiaoming Pan

**Affiliations:** 1 College of Mechanical & Electrical Engineering, Jining University, Jining 273155, China; sushen1101@163.com (S.S.); sdzluck@163.com (S.Z.); 2 College of Mechanical & Electrical Engineering, Wenzhou University, Wenzhou 325006, China; shiyuhuan0305@163.com (Y.S.); 15858827123@163.com (H.D.); 00132004@wzu.edu.cn (X.P.)

**Keywords:** selective laser melting (SLM), TC4 alloy, annealing heat treatment, microstructure, mechanical properties

## Abstract

The reduced ductility caused by the brittle needle-like α′ martensite limits the application of TC4 alloys produced by selective laser melting (SLM). Appropriate heat treatment can improve the microstructures and properties of SLM-fabricated TC4 alloys. In this work, SLM-fabricated TC4 alloys underwent stress relief annealing at 600 °C and high-temperature annealing at 800 °C. The effects of heat treatment temperature on phase composition, microstructural morphology, grain orientation, and mechanical properties were investigated. Meanwhile, the microstructural evolution and fracture mechanisms during the heat treatment process were analyzed. The results indicate that after annealing at 600 °C, the needle-like α′ phase transforms into elongated α, and nano-β phase increases. When annealed at 800 °C, the α′ phase completely transforms into a more stable lath-shaped α phase and a short rod-shaped β phase, with the nano-β phase disappearing. The texture orientation gradually shifts from <0001> towards <01-10>, where slip systems are more active. Additionally, heat treatment promotes the transition of grain boundaries to high-angle grain boundaries, thereby alleviating stress concentration and enhancing solid-solution strengthening. After heat treatment, the ultimate tensile strength of the material slightly decreases, but the elongation significantly increases. As the annealing temperature increased, the elongation (EL) improved from 5.22% to 11.43%. Following high-temperature annealing at 800 °C, necking and larger dimples appear on the fracture surface, and the fracture mechanism shifts from a mixed brittle–ductile fracture to a ductile fracture. This work provides a theoretical basis for improving the microstructures and properties of SLM-fabricated TC4 alloys through heat treatment.

## 1. Introduction

TC4 is a typical α + β dual-phase titanium alloy widely used in aerospace and other fields due to its high specific strength, excellent strength-toughness combination, and good corrosion resistance [1,2,3]. However, traditional processing methods suffer from issues such as low material utilization and difficulties in manufacturing complex components [4,5]. Selective laser melting (SLM) technology, leveraging its high energy density and fine laser spot characteristics, enables direct fabrication of complex-structured TC4 components while achieving metallurgical bonding and dense microstructure, making it a key solution to these challenges [6,7,8].

Nevertheless, the rapid heating–cooling cycles and non-equilibrium solidification inherent in SLM tend to promote the formation of fine needle-like α′ martensite in TC4 [9,10]. However, material strength could be enhanced by the α′ phase. But the insufficient ductility of the α′ phase results in materials failing to meet the comprehensive performance requirements of aerospace load-bearing components. Thus, the core challenge in this field lies in improving plasticity without significant strength loss [11,12].

Therefore, researchers have conducted extensive studies, including process optimization, alloy composition adjustment, twinning-induced plasticity enhancement, and exploration of various post-processing techniques [13,14,15]. Among these, hot isostatic pressing (HIP) and heat treatment are currently the two most commonly used post-processing methods. HIP primarily improves plasticity by closing internal pores in SLM-fabricated components [16], but its effectiveness is limited. The high processing costs, along with difficulties in meeting the requirements of complex part geometries, restrict its widespread application.

Existing research has demonstrated that heat treatment is an effective approach for regulating the microstructure and properties of SLM-fabricated TC4. The transformation of the α′ phase into α + β phases could be promoted by annealing at 650 °C, improving plasticity [17]. Complete α′ phase transformation could be achieved by treatment at 750–900 °C, increasing tensile properties by approximately 70% [18,19]. High-temperature treatments can further enhance plasticity through grain refinement or microstructural reconstruction [20,21,22,23,24]. However, the current studies have two major limitations. One is an insufficient understanding of the synergistic evolution mechanisms of microstructure and texture near the critical temperature between the α phase and α + β phase regions. The other is a lack of correlation analysis between heat treatment-induced texture transformation and plasticity improvement. Therefore, it is difficult to develop targeted performance regulation strategies.

This study focuses on addressing these research gaps by systematically investigating the effects of heat treatment at 600 °C (α-phase region) and 800 °C (α + β phase region) on the microstructure and texture of SLM-fabricated TC4. It elucidates the mechanisms by which phase composition and texture transformation enhance mechanical properties and establishes the relationship among the “heat treatment process–microstructure–performance.”7 The findings provide actionable heat treatment solutions and theoretical support for the engineering application of SLM-fabricated TC4 components.

## 2. Experimental Section

### 2.1. Materials and Processing

**Figure 1:**
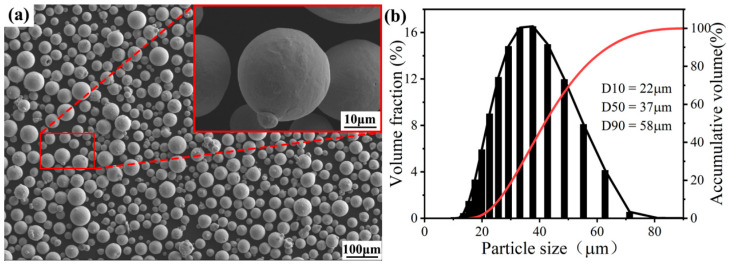
TC4 powders: (**a**) SEM image, (**b**) particle distribution [25].

Industrial TC4 powders (Xi’an Ouzhong Technology, Xi’an, China) were used in this work. As shown in Figure 1, the powder particles exhibit a spherical shape with a sphericity of approximately 0.95. They have a uniform size distribution ranging from 22 to 58 μm, and the average particle size is about 37 μm. The powder surface is smooth and free of agglomeration, with a flowability of 37.1 s/50 g, a bulk density of 2.40 g/cm^3^, and a tapped density of 2.98 g/cm^3^. The chemical compositions are listed in Table 1. Before the experiment, the powder was dried at 100 °C for 1 h. The TC4 samples were directly fabricated using an SLM system (HBD-280T, Hanbang Technology, Zhongshan, China), equipped with a 500 W continuous fiber laser (YLR-500-WC, IPG Photonics, Marlborough, MA, USA) with a wavelength of 1070 ± 10 nm. The layer thickness ranged from 20 to 100 μm, the forming accuracy was approximately 0.05 mm, and the maximum build size was 250 mm × 250 mm × 300 mm. Before fabrication, the TC4 alloy samples were designed and support was added by using 3D modeling software (Voxel Dance Additive 5.0, Shanghai Mange Technology Co., Ltd., Shanghai, China) and saved in VXD format. Then, the VXD format file was processed using slicing software (HBD Build Expert: 3.9.0.422, Hanbang Technology, Zhongshan, China) and saved in STL format. Finally, the sliced STL file was imported into the SLM system for sample fabrication based on predefined parameters.

**Figure 2:**
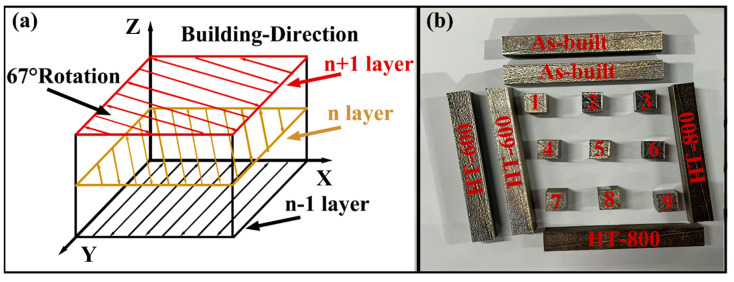
(**a**) Scan strategy, (**b**) as-built alloys.

The process parameters for fabricating TC4 alloys using SLM were as follows: laser power of 330 W, scanning speed of 1350 mm/min, scanning distance of 120 μm, spot diameter of 80 μm, and layer thickness of 50 μm. To reduce residual stress during the sample fabrication process, a zigzag scanning strategy with an interlayer rotation of 67° was employed [25], as shown in Figure 2a. High-purity argon gas (≥99.99%) was continuously supplied during the SLM process to prevent oxidation of the alloys. Figure 2b shows the nine 10 mm × 10 mm × 10 mm cubic alloys and six 10 mm × 10 mm × 100 mm horizontally constructed rectangular standard tensile specimen blanks, which were prepared for heat treatment and tensile testing, respectively.

### 2.2. Heat Treatment Processes

The heat treatment was conducted in a GSL-1500X tube furnace (Hefei Kejing Materials Technology Co., Ltd., Hefei, China). To prevent high-temperature oxidation, the samples to be treated were first sealed in a high-purity argon-filled quartz tube (Φ50 × 700 mm) and then placed in the furnace for treatment. Three groups of samples were prepared for the heat treatment experiments, with each group consisting of three cubic samples (for microstructural analysis and microhardness testing) and two tensile sample blanks (for tensile testing and fracture morphology analysis, as shown in Figure 2b). Based on references [26,27], in order to ensure that the α′ phase fully transforms into the α + β phase and to reduce residual stress, two heat treatment processes were set: HT-600 (600 °C) and HT-800 (800 °C), both of which were carried out separately. The specific parameters were as follows: heating rate of 10 °C/min, holding time of 2 h, and furnace cooling (FC) at a rate of 5 °C/min. The heat treatment processes and sample designations are detailed in Table 2.

### 2.3. Microstructure Characterization and Mechanical Test

Before and after heat treatment, the TC4 cubic alloys were ground and polished, then etched in Kroll’s reagent (17 mL H_2_O, 2 mL HNO_3_, and 1 mL HF) for microstructural observation. The microstructures were characterized using three instruments. The first was a D8 Advance XRD analyzer (Bruker, Bremen, Germany, with Cu-Kα radiation, operated at 40 kV and 15 mA, scanning angles from 20° to 80°, a step size of 0.02°, and a scan speed of 2°/min). The second was a CDM-806C optical microscope (Shanghai Tuming Optical Instrument Co., Ltd., Shanghai, China). The third was a QUANTA FEG450 scanning electron microscope (FEI, Hillsboro, OR, USA) equipped with an EBSD attachment. The microhardness of the alloys was measured using an HM-200 Vickers hardness tester (Shanghai Hengyi Precision Instrument Co., Ltd., Shanghai, China), with a load of 0.5 kg and a loading time of 15 s. The reported microhardness values are the averages of six measurements.

**Figure 3:**
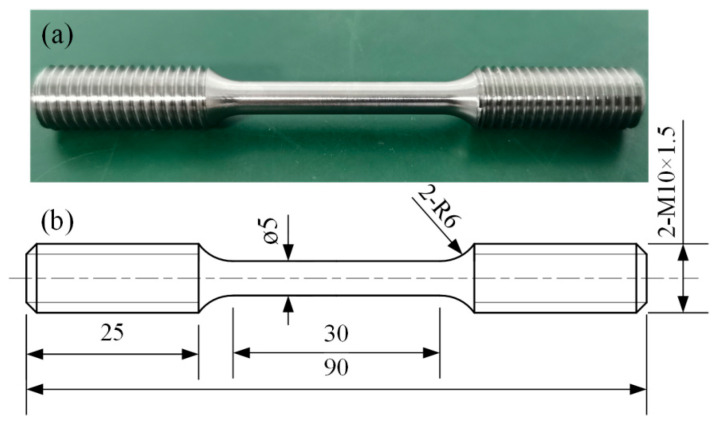
(**a**) The tensile sample and (**b**) its dimensions (unit: mm) [25].

The tensile samples shown in Figure 3 were prepared to perform turning and milling precision machining on the three groups of rectangular tensile sample blanks in Figure 2b (two in each group, including those without heat treatment, and with heat treatment at 600 °C and 800 °C), according to the GB/T 228.1-2021 standard [28] and dimensional requirements (Figure 3b). The tensile test was conducted on the C51.105 universal tensile testing machine (Shenzhen Chuyinghao Technology Co., Ltd., Shenzhen, China) at room temperature (25 °C) and a tensile rate of 1 mm/min. Each group of samples was repeated twice, and the tensile axes were all perpendicular to the construction direction. According to Formula (1) and the measurement method in GB/T 228.1-2021 standard, the elongation was calculated by measuring the length of the sample after fracture, and the fracture mechanism was analyzed in combination with the SEM characterization of the fracture surface.

A = (L − L_0_)/L_0_ × 100% (1)

## 3. Results and Discussion

### 3.1. Phase Analysis

**Figure 4:**
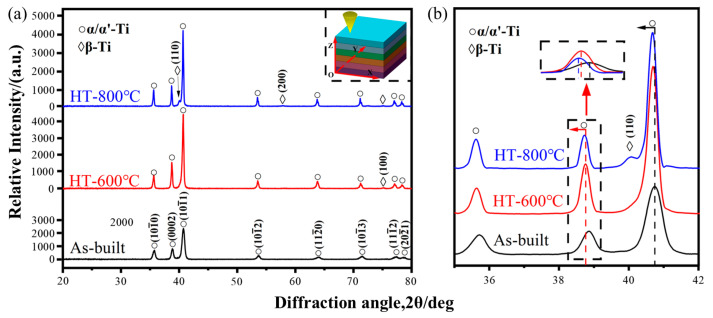
XRD analysis results: (**a**) XRD patterns of as-built and heat-treated alloys; (**b**) Partial enlargement of XRD patterns.

Figure 4 shows the XRD phase patterns of the as-built and heat-treated specimens (HT-600 and HT-800) on the XOZ cross-section within the 2θ range of 20–80°. The transformation of BCC β phase into HCP α′ phase is promoted by the rapid cooling during SLM fabrication, resulting in the as-built sample being predominantly composed of α/α′ phases. During annealing at 600 °C (HT-600), although below the critical decomposition temperature of the α′ phase (~760 °C), limited diffusion of alloying elements (Al, V, etc.) induces a diffusion-controlled α′ → α + β transformation, causing partial decomposition of metastable α′ into stable α phase and consequently increasing α phase content. This degree of transformation is governed by elemental diffusion behavior [29]. A weak β phase diffraction peak is detected near 75° in the XRD pattern, indicating that the HT-600 microstructure consists mainly of α′ phase with minor α and β phases. In the HT-800 sample, additional β phase diffraction peaks are detected at approximately 40° and 57.8°, attributable to the heat treatment temperature exceeding the α′ decomposition threshold (~760 °C), resulting in enhanced element diffusion. This enables more complete α′ → α + β transformation, yielding a final microstructure comprising primarily α and β phases with nearly complete α′ phase decomposition.

Furthermore, as depicted in Figure 4b, an increase in the heat treatment temperature results in a notable decrease in the half-width of the α/α′ phase diffraction peaks. It is also accompanied by a gradual shift towards lower angles. This phenomenon signifies that the α/α′ phases within the microstructure are undergoing coarsening and that their lattice dimensions are experiencing distortion. During the phase transformation from α′ to α, aluminum (Al) commonly functions as a substitutional solute. Specifically, the α′ phase separates vanadium (V) atoms while incorporating an increased number of Al atoms. This leads to the formation of a new α phase that is enriched with soluble Al. Given that the atomic size of Al is larger than that of V, the interplanar spacing of the newly formed α phase expands. This causes a corresponding reduction in the diffraction angle [30,31]. As the heat treatment temperature escalates, the solubility of the α phase also augments, resulting in a more pronounced shift in the diffraction peaks for HT-800. This shift not only reflects structural changes but also enhances solid solution strengthening within the alloys.

### 3.2. Microstructures

#### 3.2.1. Microstructural Morphology

**Figure 5:**
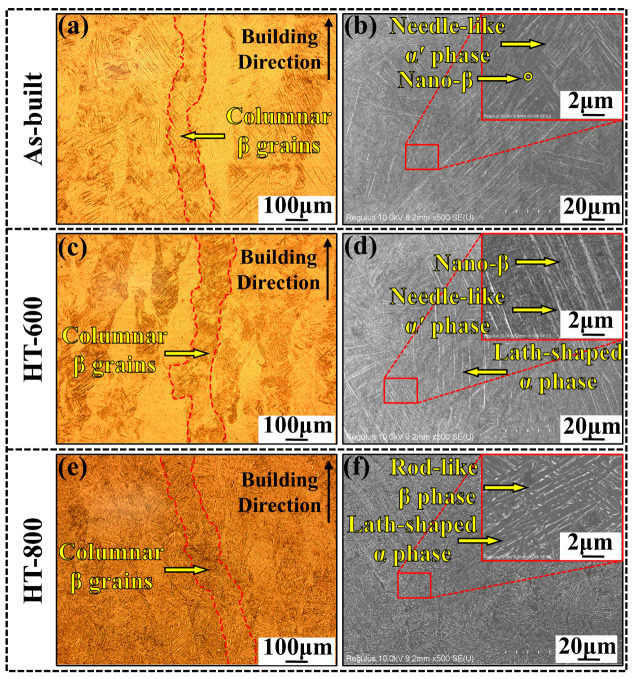
Microstructure of XOZ cross section of as-built alloy and heat-treated alloys: (**a**,**b**) as-built; (**c**,**d**) HT-600; (**e**,**f**) HT-800.

Figure 5 shows the microstructural morphology of the XOZ cross-section of the as-built alloys and heat-treated alloys (HT-600 and HT-800). All three groups exhibit columnar β grains. These grains span multiple deposited layers, extending in the direction opposite heat conduction and possessing heights within the range of 500 to 800 μm. However, no significant layering is discernible within these grains. There are no obvious differences in the morphology and width (ranging from 100 to 150 μm) of the columnar grains on the XOZ cross-section when comparing the as-built alloys to the heat-treated ones. This suggests that the heat treatment process effectively maintained the initial grain morphology of the alloys.

**Figure 6:**
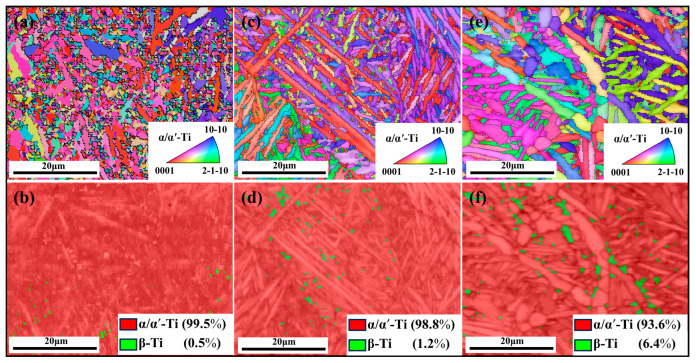
Orientation and phase maps of as-built (**a**,**b**), HT-600 (**c**,**d**), and HT-800 alloys (**e**,**f**).

Combining the OM and SEM images in Figure 5, it can be observed that in the as-built sample, the rapid cooling effect of the SLM molten pool results in the formation of numerous needle-like α′ martensite (transformed from β phase). They are distributed within the columnar β grains, with a small amount of nano-β phase (approximately 0.5%) precipitated at their boundaries (Figure 5b and Figure 6a,b). Compared to the as-built sample, the HT-600 sample exhibits α′ martensite with a smaller aspect ratio within the columnar β grains, along with a higher content of nano-sized β phase (1.2%) and lath-shaped α phase (Figure 5d and Figure 6c,d). This indicates that the 600 °C heat treatment temperature triggers the partial transformation of α′ phase into β and α phases. Therefore, the content of both phases increases in the TC4 sample, which is consistent with the XRD results. After high-temperature annealing at 800 °C, significant changes occur in the α′ phase structure. The metastable α′ phase almost completely decomposes into lath-shaped α phase and short rod-like β phase. As the temperature rises into the α + β two-phase region, the α′ phase first transforms into α phase, followed by the nucleation of the β phase at the α phase boundaries. Meanwhile, the residual β phase between α′ martensite absorbs the nucleated α′ phase and grows. As shown in Figure 5f and Figure 6e,f, the α′ phase has essentially disappeared, and the β phase aggregates and grows into short rod-like structures. It results in a microstructure consisting of lath-shaped α phase with approximately 6.4% short rod-like β phase distributed between them.

#### 3.2.2. Grain Orientation and Distribution

SLM is a rapid heating/cooling process that results in polycrystalline crystallization occurring far from equilibrium conditions. Due to the heat transfer dynamics, the grains grow with a specific degree of orientation. Furthermore, subsequent heat treatment can alter the orientation distribution of these polycrystals, leading to a texture that plays a crucial role in determining the mechanical properties of the alloys.

**Figure 7:**
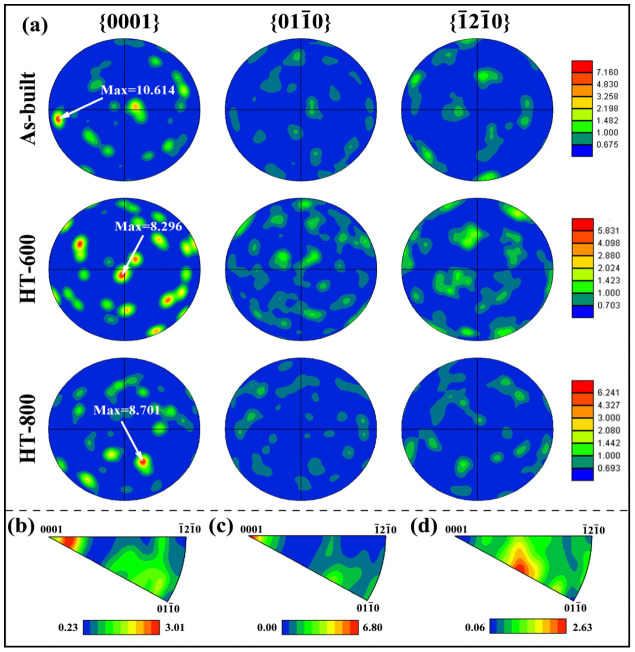
Pole figure of alloys before and after heat treatment (**a**); inverse pole figure: as-built (**b**), HT-600 (**c**), and HT-800 (**d**).

Figure 6 and Figure 7 show the grain orientation and texture distribution for the as-built alloys and the heat-treated alloys, respectively. From Figure 6, it can be observed that the grain orientation of the as-built alloys is primarily concentrated on the {0001} planes. After heat treatment, the grain orientation of the heat-treated alloys (HT-600 and HT-800) starts to shift towards the {10-10} and {2-1-10} planes (Figure 6a,c,e). Heat-treated alloys exhibit diversified orientation characteristics and a reduction in anisotropy. This is beneficial for improving the performance of the alloys. Combined with Figure 7, it is further evident that all three alloys exhibit a strong texture orientation on the {0001} planes. The as-built alloys show strong texture concentrated at both ends of the {0001} pole figure, with a maximum value of 10.614. In contrast, the two heat-treated alloys primarily show their texture distributed in the middle of the {0001} pole figure, with texture strengths of 8.296 and 8.701, respectively. This indicates that heat treatment weakens the texture of the SLM-fabricated TC4 alloy. This is because the original β grains, after undergoing high-temperature treatment transitioning from β → α′/αGB → α + β, can produce up to 12 different α′/α orientations. This significantly reduces the texture strength [32]. Additionally, after the 800 °C heat treatment, the texture distribution of the alloys moves away from the axis of the {0001} pole figure, indicating that the texture along the {0001} direction begins to gradually shift towards other directions.

In summary, the mechanical properties of the material can change significantly with variations in orientation distribution. The texture in both the as-built and HT-600 alloys is predominantly aligned along the <0001> crystallographic direction, leading to a hardness level that surpasses that of alloys featuring other orientations. Conversely, the texture orientation of the HT-800 alloys exhibits a shift towards the <01-10> crystallographic family (Figure 7d). This shift is significant because the slip systems within the <01-10> crystallographic family are more active, facilitating deformation within the material and consequently enhancing its plasticity.

#### 3.2.3. Grain Boundary Distribution and Dislocation Density

**Figure 8:**
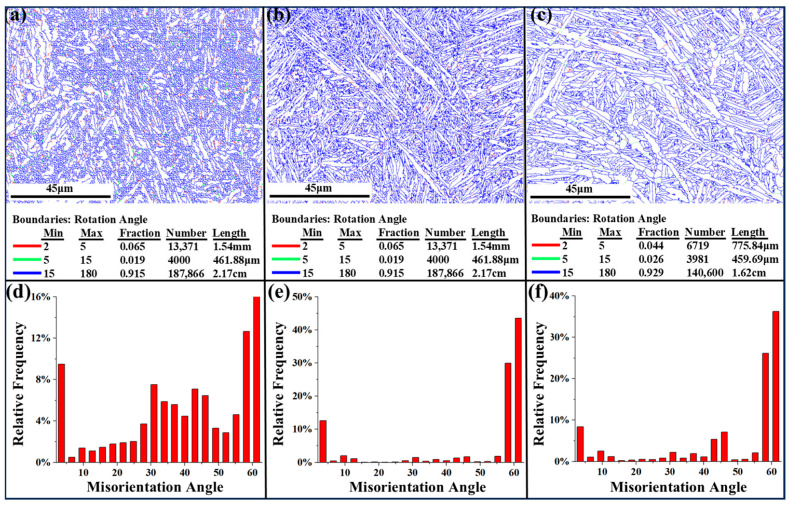
Grain boundary misorientation maps and angle distribution in the as-built (**a**,**d**), HT-600 (**b**,**e**), and HT-800 (**c**,**f**) alloys.

**Figure 9:**
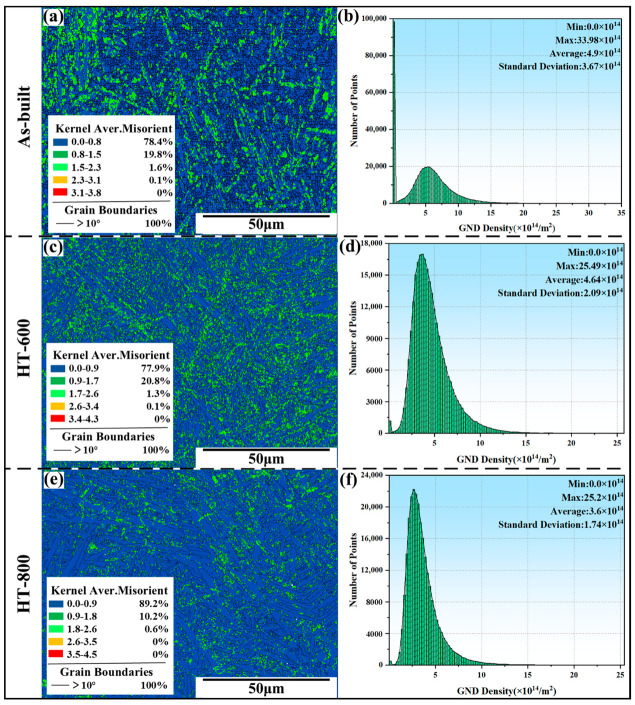
KAM and GND density distribution maps: (**a**,**b**) as-built, (**c**,**d**) HT-600, and (**e**,**f**) HT-800 alloys.

Figure 8 and Figure 9 illustrate the grain boundary and kernel average misorientation (KAM) distributions for the as-built and heat-treated alloys, respectively. Figure 8 reveals that the grain boundaries in the as-built alloys are closely packed. This is attributed to the abundance of needle-like α/α′ phases. After heat treatment, the microstructure coarsening results in a less dense grain boundary distribution in the heat-treated alloys. Furthermore, Figure 8d,e,f illustrates that the angle distribution in the as-built alloys is relatively uniform. In contrast, the angles in the two heat-treated alloys are predominantly focused on high-angle grain boundaries (HAGBs) exceeding 55° and low-angle grain boundaries (LAGBs) below 5°. This aligns with the XRD patterns in Figure 3, indicating that a high-temperature treatment leads to a prominent presence of <1120>α, characterized by a 60° orientation difference within the original β grains [33]. Consequently, the angles shift towards HAGBs following heat treatment. According to the Arrhenius equation [34], high heat treatment temperatures facilitate the transformation between LAGBs and HAGBs, resulting in some HAGBs converting to LAGBs.

From Figure 9a,c, it is apparent that the as-built and HT-600 alloys exhibit a relatively high proportion of large KAM values, with regions where KAM surpasses 2.6°. This indicates that these two groups have considerable grain orientation differences, potentially leading to stress concentration and limiting the material’s capacity for local deformation. Correspondingly, Figure 9b,d show that the as-built and HT-600 alloys have a high geometrically necessary dislocation (GND) density, indicating pronounced stress concentration and a high density of dislocations in localized regions. This indicates that more defects appear within the crystal structure. When external forces exist, these areas are prone to preferentially deform, serving as initiation points for crack propagation and ultimately resulting in reduced toughness. In contrast, following the 800 °C high-temperature annealing treatment, not only is the issue of internal stress concentration mitigated (Figure 9e), but the problem of high dislocation density is also alleviated (Figure 9f), thereby enhancing plasticity. This contributes to reducing the risk of premature failure in spacecraft load-bearing components during operation, thereby enhancing their reliability and long-term stability under complex alternating loads.

### 3.3. Mechanical Properties

#### 3.3.1. Microhardness

**Figure 10:**
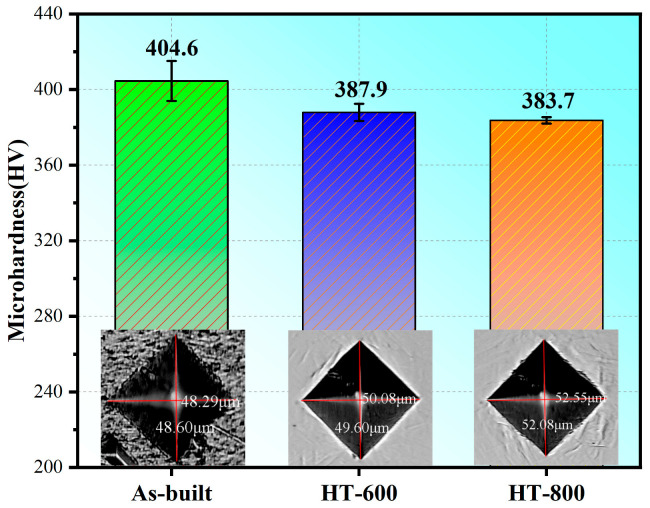
Microhardness of as-built and heat-treated alloys.

Figure 10 shows the microhardness of the as-built and heat-treated alloys. In comparison to the as-built alloys (404.6 HV), the microhardness of the HT-600 alloys (387.9 HV) undergoes a decrease of approximately 4%. This reduction is attributed to the substantial alleviation of residual stress and stress concentration issues accumulated during the SLM process following the 600 °C stress-relief annealing, coupled with the precipitation of nano-β phases. Upon undergoing 800 °C high-temperature annealing, the microhardness decreases further by roughly 5.1%. This decline can be attributed to two factors. Firstly, high-temperature annealing effectively removes residual stress within the microstructures, thereby reducing stress concentration. Secondly, the metastable hard phase α′ nearly completely decomposes. This leads to the coarsening of lath-shaped α phases and the formation of short rod-like β phases, accompanied by a shift in grain orientation from <0001> to the more active slip system <01-10>. The HT-800 alloys exhibit the smallest standard deviation in microhardness among the three groups, signifying a more uniform microhardness distribution on the surface after 800 °C annealing. This uniformity can be correlated with the significant reduction in GND values (Figure 9), which to some extent mitigates internal stress concentration issues.

#### 3.3.2. Tensile Property and Fracture Mechanism

**Figure 11:**
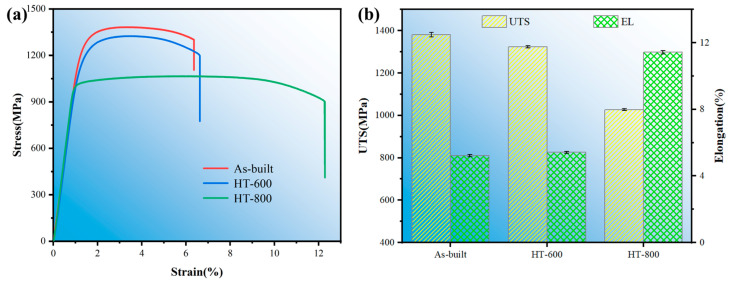
Tensile properties of as-built and heat-treated alloys: (**a**) stress–strain curve, (**b**) ultimate tensile strength and elongation.

Figure 11 delineates the tensile properties of both the as-built and heat-treated alloys. Specifically, Figure 11a depicts the stress–strain curves of the alloys, whereas Figure 11b shows their ultimate tensile strength (UTS) and elongation (EL). According to the figures, the as-built alloys exhibit a UTS of 1381 MPa, with a fracture elongation of 5.22%. After annealing at 600 °C, the UTS undergoes a slight decrement. However, the increase in EL remains insignificant. Conversely, after undergoing high-temperature annealing at 800 °C, the UTS experiences a more pronounced drop, whereas the EL experiences a substantial enhancement, surging from 5.22% in the as-built alloys to 11.43%. The HT-800 sample meets the tensile property requirements (tensile strength ≥895 MPa, elongation ≥10%) for Grade F-5 titanium alloys as specified in ASTM B381-13(R2019) [35], making it suitable for manufacturing spacecraft load-bearing components.

The decrease in the ultimate tensile strength (UTS) of the alloys correlates with the content of needle-like α′ martensite, whereas the increase in fracture EL is related to the proportions of α and β phases. As discussed in Section 3.2, annealing at 600 °C results in partial decomposition of the hard α′ martensite in the microstructure of the HT-600 alloys. This leads to a reduction in α′ martensite content and a corresponding slight decrement in UTS. Simultaneously, the contents of α and β phases increase, with the β phase initiating aggregation and growth. However, the texture remains predominantly aligned in the <0001> direction. This contributes to only a minor enhancement in plasticity. Following high-temperature annealing at 800 °C, residual stresses within the microstructure are largely alleviated, mitigating stress concentration. Furthermore, the near-complete disappearance of α′ martensite, coupled with a substantial increase in both α and β phases, results in the β phase aggregating and growing into short rod-like structures. Additionally, the texture begins to shift towards the <01-10> direction. These combined factors contribute to a decline in UTS while significantly augmenting plasticity. The enhanced plasticity resulting from texture reorientation toward active slip systems significantly improves the material’s adaptability under stress conditions, which is of critical importance for enhancing the service reliability of spacecraft load-bearing components.

**Figure 12:**
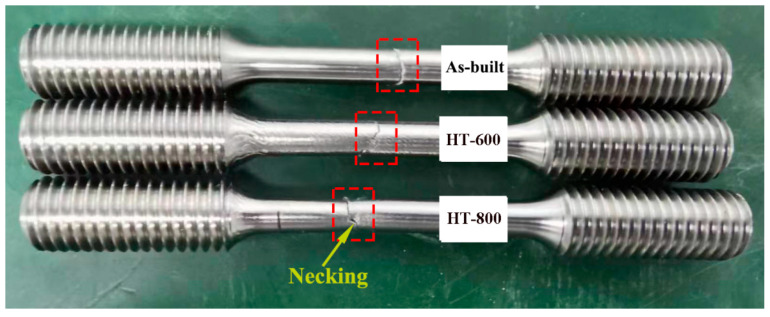
Macroscopic fracture morphology of alloys.

**Figure 13:**
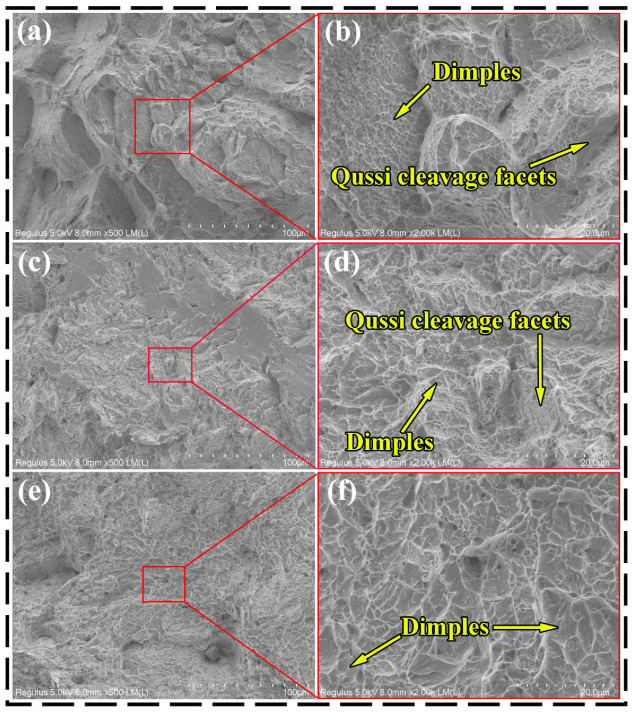
Microscopic fracture morphology of the as-built (**a**,**b**), HT-600 (**c**,**d**), and HT-800 (**e**,**f**) alloys.

Figure 12 and Figure 13 illustrate the macroscopic and microscopic fracture morphologies, respectively, of the as-built and heat-treated alloys. As depicted in Figure 12, the fractures of both the as-built and HT-600 alloys display a lack of significant plastic deformation, with a reduction in area of less than 5%, suggesting brittle fracture characteristics. In stark contrast, the HT-800 alloys exhibit evident necking at the fracture site, accompanied by a reduction in area exceeding 5%. Furthermore, a notable elongation of the tensile samples, compared to the previous two groups, indicates ductile fracture behavior. From the microscopic fracture morphology of the tensile samples presented in Figure 13, it is evident that the fractures of both the as-built and HT-600 alloys feature a mixture of stepped cleavage and equiaxed shallow dimples. They highlight a mixed fracture mechanism that embodies both brittle and ductile attributes, albeit to varying degrees. Conversely, the fracture surface of the HT-800 alloys, which undergoes high-temperature annealing, presents a distinctive fibrous appearance. It is adorned with numerous larger and deeper dimples, indicative of a mechanism predominantly governed by ductile fracture.

Based on the microstructure analysis conducted in Section 3.2, there exists no significant divergence in the fracture mechanisms between the HT-600 and as-built alloys. This similarity stems from the fact that stress-relief annealing at 600 °C results in only partial decomposition of the hard α′ martensite within the alloys. The texture orientation predominantly remains the low-ductility <0001>. Consequently, there are no notable differences in microstructures and compositions between the two. In stark contrast, high-temperature annealing at 800 °C accelerates the decomposition of the hard α′ martensite, causing the needle-like α′ phase to nearly completely transform into lath-shaped α and short rod-like β phases. This transformation leads to a transition from a Widmanstätten structure to a basketweave structure, which significantly enhances ductility. Additionally, the texture orientation gradually shifts from <0001> to <01-10>. Together, these alterations improve the material deformability, resulting in a fracture mechanism primarily characterized by ductile failure. Thus, compared to annealing treatments below the α′ decomposition temperature, annealing at 800 °C within the (α + β) phase region markedly enhances ductility. This improvement is primarily attributed to the disappearance of the brittle α′ phase, the increase in the ductile β phase, and the texture transition towards the <01-10> direction. Therefore, the 800 °C high-temperature annealing process can significantly enhance both ductility and fracture toughness while maintaining material strength, making it particularly suitable for manufacturing critical load-bearing connectors in spacecraft that undergo tensile stresses. This advancement further expands the application potential of TC4 alloy in high-performance aerospace components.

## 4. Conclusions

This study investigates the regulation of microstructure and properties in SLM-fabricated TC4 alloy through heat treatment processes, elucidating the influence of heat treatment temperature on phase composition, microstructure, texture, and mechanical properties. The mechanisms of microstructural evolution and fracture behavior during heat treatment are systematically analyzed. This study provides both theoretical and technical support for the performance optimization of TC4 alloy in engineering applications. The main conclusions are as follows:

(1) Microstructure control mechanism: The SLM microstructure of TC4 is mainly needle-like α′ martensite, with a small amount of nano-β phase (volume fraction 0.5%) distributed at the boundary, and the texture is mainly <0001>. The decomposition of the α′ phase part into lath-shaped α and nano-β phases and an increase in the β phase content are triggered by annealing at 600 °C. After heat treatment at 800 °C (above the α′ decomposition temperature of 760 °C), the brittle α′ phase almost completely transforms into stable lath-shaped α and short rod-like β phases. Meanwhile, the volume fraction of the β phase increases to 6.4%. The texture shifts towards the <01-10> direction, where the slip system is more active, achieving the regulation of the microstructure from being brittle-dominant to being tough-dominant.
(2) Performance optimization rule: The tensile strength of the SLM-formed TC4 reaches 1381 MPa, but the elongation is only 5.22%. After heat treatment at 600 °C, the performance changes are relatively small, and the fracture is still mainly a mixture of brittleness and toughness. After heat treatment at 800 °C, the strength decreased by 18% (to 1027 MPa), but the elongation increased to 11.43%, achieving a balance between strength and plasticity. It meets the requirements of grade F-5 in ASTM B381-13 (R2019) standard. The fracture mechanism is transformed into a ductile fracture. This optimization stems from the synergistic effects of the α′ → α + β phase transition in reducing brittle phases, β phase toughening, and texture optimization.
(3) Engineering guidance value: This study clarifies the quantitative influence of heat treatment temperature on the microstructure and properties of SLM-fabricated TC4. Meanwhile, it is proposed that the performance could be further optimized by regulating the holding temperature (such as optimizing the β phase content at 800 °C) and the cooling rate (refining the α/β phase distribution). A basis is provided for heat treatment process design for applications in load-bearing components in aerospace and other fields. However, the heat treatment temperature range of this study is limited (it does not cover higher temperatures or more temperature values). Additionally, the performance is only evaluated through “horizontal” stretched parts (with the length direction perpendicular to the forming direction), without a comprehensive analysis of the tensile performance in different directions. There are certain limitations. In the future, systematic research will be carried out on the above aspects.

## Figures and Tables

**Table 1:** Chemical compositions of TC4 powders (wt.%) [25].

Elements							
Al	V	Fe	C	N	H	O	Ti
5.5–6.5	3.4–4.5	≤0.25	≤0.08	≤0.03	≤0.012	≤0.1	Bal

**Table 2:** Heat treatment process and the abbreviation for alloys.

Sample	Temperature (°C)	Time (h)	Cooling
As-built	None	None	None
HT-600	600	2	FC
HT-800	800	2	FC

## Data Availability

The original contributions presented in this study are included in the article. Further inquiries can be directed to the corresponding authors.
